# Low-threshold support for families with dementia in Germany

**DOI:** 10.1186/1756-0500-5-317

**Published:** 2012-06-21

**Authors:** Iris Hochgraeber, Sabine Bartholomeyczik, Bernhard Holle

**Affiliations:** 1 German Center for Neurodegenerative Diseases (DZNE), Witten, Germany; 2 School of Nursing Science, Faculty of Health, Witten/Herdecke University, Witten, Germany

**Keywords:** Dementia, Study protocol, Community care service

## Abstract

**Background:**

Low-threshold support services are a part of the German health care system and help relieving family caregivers. There is limited information available on how to construct and implement low-threshold support services for people with dementia and their families in Germany. Some studies describe separately different perspectives of experiences and expectations, but there is no study combining all the different perspectives of those involved and taking the arrangements and organisation as well as their opinions on supporting and inhibiting factors into consideration.

**Findings:**

This protocol describes the design of the study on low-threshold support services for families with a person with dementia in two German regions. The aim is to develop recommendations on how to build up these services and how to implement them in a region. A quantitative as well as a qualitative approach will be used. The quantitative part will be a survey on characteristics of service users and providers, as well as health care structures of the two project regions and an evaluation of important aspects derived from a literature search. Group discussions and semi-structured interviews will be carried out to get a deeper insight into the facilitators and barriers for both using and providing these services. All people involved will be included, such as the people with dementia, their relatives, volunteers, coordinators and institution representatives.

**Discussion:**

Results of this study will provide important aspects for policymakers who are interested in an effective and low-threshold support for people with dementia. Furthermore the emerging recommendations can help staff and institutions to improve quality of care and can contribute to developing health and social care structures in Germany.

## Findings

A recently published study about the care situation in Germany focussing dementia shows that the number of people in need of care has reached a new high with over 2 million people [1]. Also the number of people with dementia is growing [1]; one third of men and half of the women will experience dementia during their lifetime [1]. During the course of the disease people with dementia nearly always need care with 30 to 60% living at home. Due to an increasing shortage of professional staff informal support becomes more and more important [2]. Family caregivers have an important role in keeping people with dementia in the community which is a crucial factor in health care policy and an aim of German Long Term Care Insurance (LTCI) “outpatient before inpatient” [3].

## Low-threshold support services in Germany

The German Ministry of Health describes low-threshold support services as a “type of service in which helpers [volunteers] provide care for persons in need in group situations or at home, and support relatives by relieving and advising them.” [3]. If persons’ activities of daily living are assessed as permanently limited, they receive up to 200€ a month reimbursement from the insurance [4]. These people are mainly persons with cognitive impairment and dementia. The 200€ are intended to be used for low-threshold support services. One hour of low-threshold support costs between 5 and 20 Euros [5].

Low-threshold support services can be divided into group services, like social care groups, and individual respite care provided by volunteers in clients’ homes. Usually this involves social activities rather than care. This type of service is called home social care for the purpose of this study. Even though, the volunteers have no special dementia and nursing education, they do receive between 20–40 hours of training. The services are coordinated and organised by professionals such as nurses or social workers, who also supervise the volunteers. Rather than being provided for longer periods of time, like months or weeks, the care is offered on an hourly basis.

Further characteristics for so-called low-threshold services are: a nearby location, flexibility and affordability [6]. Other services like counselling and support groups for relatives are also included in this kind of service but they are not considered in this study. In this study, the focus is on services where care is provided for people with dementia.

It is not known exactly how many services exist in Germany at present. However, a list was published containing 755 approved low-threshold support services in North-Rhine Westphalia in 2009 [7]. A service only gets financial support if official approval has been issued.

The number of services which have not been approved but are also used by people in need of care is unknown. Also unknown is the number of services especially for people with dementia. However, it can be assumed that most users of approved low-threshold support services are people with dementia because people with dementia will be (partly) reimbursed for their service utilization. The services are often incorporated in a welfare organization or other care institution. These organizations offer facilities, organizational structures and training for the volunteers.

Families with a person with dementia utilizing low-threshold support services are few in numbers [8,9]. A study of Gräßel *et al.*[10-12] shows that of 404 family caregivers there were 43% who knew about social care groups and 52% who knew about home social care services while the utilization rate for social care groups was 12% and for home social care services 18%. Another German study found that low-threshold support services were used by 28% of the caregiving women and by 23% of the caregiving men [13]. Furthermore, Toseland *et al.*[14] found out that enabling variables such as “knowledge of and barriers to service use”, “availability of health insurance” and “transportation” explain more variance in utilization than do need (problem/challenging behaviour of care recipient, caregiver’s burden, functional status of care recipient, stage of dementia *etc.*) or predisposing variables (satisfaction with service use, caregiver/care recipient relationship, demographics). Toseland´s *et al.*[14] enabling factors are similar to low-threshold characteristics as described earlier.

The amount of scientific literature in Germany concerning low-threshold support services is very limited, although the number of services grows continuously. There are several reports regarding the implementation of these services [15,16], descriptions of the volunteers’ perspective [17] and concepts based on experiences how to train volunteers [18,19]. Some general recommendations on how to identified social care groups [20,21] and home social care or care by volunteers [5] have also been published. Furthermore, some theses and dissertations as well as two studies were found which focussed on low-threshold support services each from a single perspective [6,10,11,22-27]. No study has yet been published which investigates low-threshold support services to answer the question how they should be constructed and combining all perspectives involved, these being: people with dementia, their relatives, volunteers, coordinators and institution representatives.

The international literature is also quite limited because German low-threshold support services can hardly be compared with international services like respite care or home help [28,29], for example. Respite care in fact has the same aim, providing relief for the family caregiver, and is also described quite similarly: “Respite care is the temporary provision of care for a person with dementia at home or in an institution by people other than the primary caregiver.” [29]. However, the term is also used to describe a wide range of services with several dimensions. The place can be the home, a day-care centre or a residential setting. It can be provided by trained, untrained staff or volunteers for varying length of time ranging from hours to weeks. It can be planned or unplanned, involving also an overnight stay [29]. Zarit, Gaugler and Jarrot [30] describe in-home respite also as house-cleaning or bathing the clients. “Respite workers may be companions, homemakers, home-health aides or nurses.” [30]. The German low-threshold support services are carried out by volunteers supervised by professional staff who also plan the service which is offered on an hourly basis regularly or irregularly. Untrained companions or homemakers are not involved.

In conclusion, German low-threshold support services can be described as a possibility to relieve family caregivers and to provide meaningful activities for people with dementia, and as a service to comply with the “outpatient before inpatient” demand [3] which constitutes the main aim of the German LTCI.

## Aim and research questions

The aim of this study is the development of recommendations for the organisation and implementation of low-threshold support offers on a scientific basis. We want to identify inhibiting and supporting factors for the development and the implementation of those services. Therefore we also want to describe the situation of service providers and users.

The main research questions are:

· What is necessary for a needs-based development of low-threshold support services from the different perspectives of the involved stakeholders?

· What are inhibiting and supporting factors for the implementation from the different perspectives of the involved stakeholders?

## Methods/design

The research questions afford a deep insight into the different perspectives involved. Therefore, we decided to combine quantitative and qualitative research methods. The quantitative part puts the emphasis on the description and evaluation of the situation with low-threshold support services in the project regions and aims at research question one. The focus in the qualitative part is on the detailed description of special points of the quantitative results and it aims at finding new aspects which have not yet been mentioned. Furthermore, we want to identify inhibiting and supporting factors for the implementation (question two). The methods complement each other and therefore we expect to get a comprehensive view on the situation.

**Figure 1:**
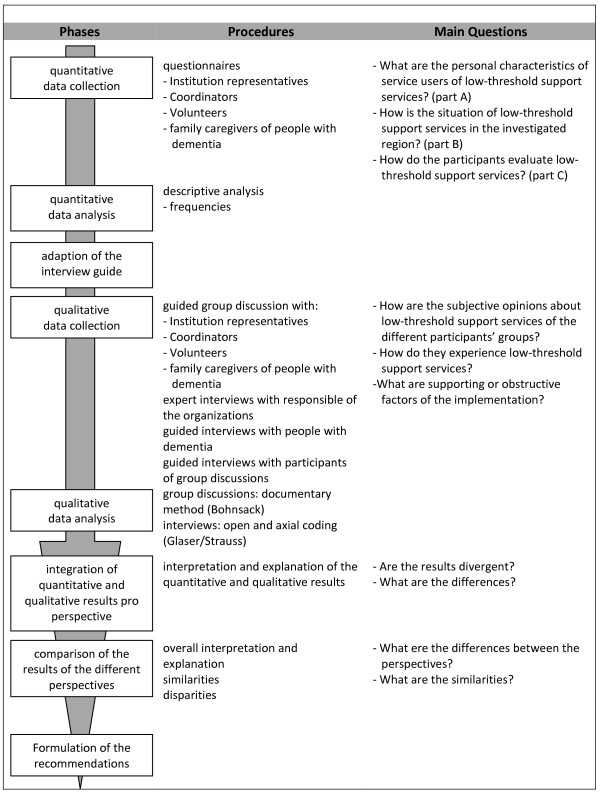
Flow chart of the study design.

The underlying approach is the action theory as described by Prein, Kluge and Kelle [31]. The authors describe two parts as fundamental. Firstly, social context conditions which structurally influence behaviour need to be identified. Secondly, it is necessary to find out how the involved people interpret these objective findings subjectively and how they react in their daily life [31,32]. Figure 1 provides an overview of the approach. The first row shows the eight phases of the study design. The second row specifies the different procedures and the third one specifies the main questions for the different study parts.

## Quantitative part of the study

The quantitative part will be a survey using a questionnaire for each perspective (family caregivers, volunteers, coordinators and institution representatives). Each questionnaire is divided into three parts. Part A describes the personal characteristics (Table 1), part B the health care structure in the region (Table 2) and part C is an evaluation of their importance using a four-point rating scale based on aspects found in the literature about low-threshold support (Table 3). This scale will not be used for determine any cut-off but only for a description of the situation. The questionnaires were developed based on a literature search and were discussed extensively with researchers experienced in surveys (face validity). The dimensions found in the literature and covered in the questionnaires were: knowledge, supervision, education, trustworthiness, reward, competences, selection (volunteer), accessibility, flexibility, continuity, costs, meal, concept of care, control, contact persons, individuality of care (service), safety of the person with dementia, relationships, competences of the coordinator [5,20,24,33]. Care here means all activities like occupation, but also physical support of the person with dementia.

**Table 1:** Questionnaire dimensions of part A

**Dimension**	**Items**	**FCG/PWD**^**1**^	**V**^**2**^	**C**^**3**^	**IR**^**4**^
**Coordinators characteristics**	Age/Gender/Educational level			X	
	Professional qualifications			X	
	Activities in direct care			X	
	Length of activities as coordinator			X	
**Volunteers characteristics**	Age/Gender/Educational level/Employment		X		
	Caring experience		X		
	Caring experience with people with dementia		X		
	Compatibility of voluntary engagement and family/job		X		
	Motivation for voluntary engagement		X		
**Family caregivers characteristics**	Age/Gender/Educational level/Employment	X			
	Relationship to care recipient	X			
	Length of caring	X			
	Support from others	X			
	Burden of caring	X			
**persons with dementia characteristics**	Age/Gender/Educational level	X			
	Care level/Problems in daily life	X			
	Diagnosis “dementia”	X			
	First memory problems	X			

^1^FCG – family caregiver; PWD– person with dementia.

^2^ V – volunteer.

^3^ C - coordinator.

^4^IR– institution representative.

**Table 2:** Questionnaire dimensions of part B

**Dimension**	**Items**	**FCG/PWD**	**V**	**C**	**IR**
**Institution characteristics**	Kind of institution				X
	Different kinds of services offered by the institution				X
	Care recipients per single service				X
	Staff members (overall)				X
	Low-threshold support offers				X
	Care recipients in low-threshold support offers				X
	Financing				X
	Aims of low-threshold support offers	X	X	X	X
**Service characteristics**	Kinds of service involved	X	X	X	
	Kinds of service offered				X
	Number of care recipients per service			X	
	Number of volunteers per service			X	
	Length of service use	X			
	Length of acting		X		
	Regularity of service use	X			
	Weekly expenditure of time		X		
	Reason for service use	X			
	Duration of care	X			
	Taking notice of the service	X	X		
	Place of service	X			
	Costs and Invoicing	X	X	X	
	Carpool			X	
	Compensation for expenses		X	X	
	Benefit by the service/use of other services	X			
	Personal benefit/Burden of the commitment		X		
	Activities offered		X		
	Extraction of volunteers			X	

**Table 3:** Questionnaire dimensions of part C

**Dimension**	**Items**	**FCG/PWD**	**V**	**C**	**IR**
**Volunteer**	Knowledge	X	X		
	Supervision		X	X	
	Education		X	X	
	Trustworthiness	X	X	X	
	Reward	X	X	X	
	Personal aptitude/competence	X	X	X	
	Selection			X	
**Service**	Accessibility	X	X		
	Flexibility of care	X	X	X	
	Continuity of care	X	X	X	
	Costs of care	X	X	X	
	Meal	X			
	Concept for care		X	X	
	Control of care	X	X	X	
	Contact person for the FCG/PWD	X	X	X	
	Individual care/adaption	X	X	X	
**Others**	Safety during care	X			
	Relationship between V and FCG/PWD	X	X	X	
	Competence of the coordinator			X	

At the beginning of the study, the questionnaires will be pretested with regard to participants’ understanding of the questions, instructions and response options as described in the example of Gorecki, Lamping, Nixon, Brown and Cano [34]. Furthermore, we want to determine whether readability is appropriate for the participants and confirm completeness of the concepts. Therefore, using a cognitive approach, we will conduct interviews for the different perspectives (family caregivers, volunteers, coordinators and representatives of the institutions). Due to the descriptive approach of the quantitative analysis, we do not see the necessity of a comprehensive statistical validation process. However, for a further use of the questionnaires this is inevitable.

## Study population and dissemination of the questionnaires

All institutions providing low-threshold support services in the two project regions will be invited to participate in the survey. The next step will be to present the project at regional healthcare conferences, where usually all the institutions are represented. Project region 1, the Heinsberg district, has a population 258,000 people living in ten communities and nine low-threshold support services, according to local descriptions. This region has a rural structure. Project region 2, the Ennepe-Ruhr-Kreis district, has about 331,000 people living in nine cities and the structure is more urban. According to a current list there are 33 low-threshold support services, of which 18 are care services exclusively for people with dementia or general services where people with dementia are included [7]. There are no official descriptions of the number service users. We will include services in rural and urban settings. We have contacts to gatekeepers in the two project regions who are involved into the regional policy of elderly care. We will call the institutional representatives in advance for having an approximately number of the different questionnaires and after a personal appointment we will provide them the questionnaires. They will disseminate the questionnaires. With this procedure we expect a high response rate. The volunteers or the coordinators will give some background information regarding the study when handing out the questionnaires. The questionnaires will be accompanied by a prepaid self-addressed envelope which is expected to ensure a high response rate.

## Analysis

Descriptive statistical analyses will be carried out to describe the frequencies of personal characteristics of the involved persons (part A), structural data (part B) and evaluation (part C). Furthermore, data will be compared according to the questions: Which characteristics do users of low-threshold support services have and what kind of services do they use? How do institutions offering low-threshold support services differ and how is the general situation of these services? Do the evaluations of characteristics of low-threshold support services differ between the different perspectives?

## Qualitative part of the study

The qualitative data collection will be conducted after the survey. In accordance with the research questions, two qualitative methods have been chosen (group discussions, guided interviews). The group discussions will be carried out with people with dementia, family caregivers, volunteers and coordinators. In addition, guided expert interviews with institution representatives and guided interviews with people with dementia using home social care are planned. The aim is to get an in-depth insight into the subjective experience with low-threshold support. We want to know what people with dementia and family caregivers think about the services they use, what problems as well as positive aspects they see. Furthermore, we want to find out how the providers (institutional representatives, coordinators) organize the services and how volunteers experience the care for people with dementia and low-threshold support services. Also, two different approaches of data analysis (documentary method [35], coding according to the Grounded Theory [36]) will be used.

## Study population and recruitment

We want to contact the qualitative study population in two ways. Firstly, the introduction of the questionnaire contains an invitation to a group discussion. Secondly, we will address potential participants directly. The cooperating institution will be highly involved in this process. Therefore, good and intensive cooperation with the institutions and explicit information beforehand are necessary. We aim to conduct two group discussions per perspective (coordinators, volunteers, people with dementia, family caregivers), about four expert interviews and four to five guided interviews with people with dementia. Furthermore, additional interviews with individual people from the group discussions are planned separately to focus on special themes.

Family caregivers and people with dementia will be assessed as to whether they are afflicted by dementia or not by the coordinators or volunteers who know the people. Therefore we will also include people without a medical diagnosis of dementia and their relatives. People with dementia who are not able to communicate anymore have to be excluded.

## Group discussions

The first group discussions focus on the perspective of coordinators of low-threshold support services, volunteers, family caregivers and people with dementia using group services. They are oriented on the model of collective framework of orientation [37,38]. The aim is, therefore, to find out the opinion of the entire group; the discussion does not focus on single opinions. There will be two kinds of groups, one consists of people who know each other from former meetings in the same group but for another purpose (volunteers, people with dementia) and the second will be groups who do not necessarily know each other but who have common experiences (family caregivers, coordinators) [35,37]. A moderator will coordinate the discussion and an assistant will be present to write down important notes. Both will also write postscripts after each discussion. The discussion will be led using a flexible guide. The topics are different for each different perspective. Themes for the coordinators are recognition, invoicing, instructions, supervision of volunteers and competences of coordinators. For the volunteers the topics are experience in caring, problems with job, family and voluntary engagement, attributes of volunteers, low-threshold care, reimbursement, burdening aspects. The guide for the discussion with family caregivers includes relief through the service, problems during care, contact person, attributes of volunteers, motivation for using the service, availability of the service, organisation of the service, invoicing. Themes for the people with dementia are experience in the group, staff member, activities, knowing each other, drop-out of group members. The guides will be used flexibly and openly to ensure a smooth discussion between the participants.

## Analysis

All group discussions will be taped and transcribed literally. Together with the postscripts the transcripts will be analysed using the “documentary method” [39]. There are different steps described by Bohnsack [35,40]. The first is the formulating interpretation where the transcripts are divided into themes and subthemes. The reflecting interpretation is the second step. A reference frame will be created by contrasting and comparing cases. Afterwards case analysis will be conducted where the results of step one and two will be connected and imbedded into the context. The last step is the typification where the results will be put into contrast so that they oppose each other. This process will be accompanied by memos. Memos are all notes taken during all phases of analysis (passages in the text, codes, feelings and thoughts). They are used to get an analytical distance to the collected data but also to stimulate creativity [41].

## Guided interviews

The interviews will be guided expert interviews [42]. The experts will be institution representatives, defined according to Meuser & Nagel [43]. That means experts are people who have responsibility for concepts or implementations or who have access to information about people involved and about decision processes. The authors differentiate expert knowledge into procedural knowledge, interpretative knowledge and contextual knowledge. In the organization part of this study the research interest is in that knowledge area. Therefore topics for the guidelines are: advantages and disadvantages of low-threshold support services relating to the organisation, authorization of services, benefits for users, barriers for service use, financing, invoicing, competences of coordinators, personal suitability of volunteers.

The second kind of interview is the guided interviews [44] with people with dementia who use home social care. The interview topics are: well-being, how the visits from volunteers are experienced, activities, determination of activities, fun, conversations, help provided by the volunteer, acceptance of the volunteer, relationship between the volunteer and the relative.

It is also planned to interview more people from the group discussion in additional individual interviews.

## Analysis

The interviews will be coded according to open and axial coding of the Grounded Theory. The Grounded Theory generates data of the natural setting and develops strategies for handling challenges [45]. Social processes and social structures from the perspective of the individual constitute the theoretical background [46]. This kind of coding seems adequate for the theoretical background. It is open and could produce new aspects from the transcripts. Within open coding, passages are separated and given a title [45]. *In-vivo*-codes are appropriate and keep the proximity to the data. Thus more abstract terms will be found and conceptualized. Afterwards the concept will be classified. Axial coding is used to find the connections between the categories and subcategories. During the coding process “comparing” and “questioning” is crucial.

## Integration of the results and the development of recommendations

The recommendations are based on the combination of quantitative and qualitative results [47]. Data sets are analysed as described before within the single perspective and qualitative and quantitative results are compared. The results can be convergent, divergent or complementary [32]. We expect complementary results in such a way that the quantitative data can be enriched by the qualitative data. Therefore the general results of the survey can be explained in depth by the qualitative results. We also expect the qualitative results to identify interpretative frameworks and emphases.

The next step will be the integration and comparison of the perspective with the focus on similarities and differences between the perspectives. Then, we will develop recommendations on how low-threshold support services should be constructed and implemented in a region. The focus will be on the practical realisation of the services and will take the results into account. The finished recommendations will be presented at the healthcare conferences in the two project regions in order to get validation of the results and feed-back.

## Ethical considerations

The ethical committee of the German Association of Nursing Science approved the study proposal and gave its clearing. For the quantitative part of the study, information is included in the letters covering the questionnaires. Furthermore, it is noted that the return of the questionnaires is by choice with participation being voluntary and completely anonymous. The group discussions and other interviews require an informed consent. Therefore we developed for every participant group an information paper and a consent paper which has to be signed by participants. For the people with dementia the language of the papers is simplified. Consent should also be given by a legal representative.

During the group discussion and interviews with the people with dementia the so-called ongoing consent [48] will be used. Here the researcher will ascertain the consent by considering the verbal and non-verbal behaviour. If there are signs of withdrawal, the researcher will stop the interview. During the group discussion a volunteer will be available to take care of people who do not want to continue the discussion. In addition, the researcher will not use words such as “dementia”, “care or support service” or other terms which impart a feeling of inferiority. Instead the researcher will adapt her language to the language of the people with dementia. Reid *et al.*[48] describes this process as “*[…] meet people on their own terms*”. The interview atmosphere should be comfortable and the duration will be as short as possible.

## Discussion

This manuscript describes the design of a study focussing on low-threshold support services in two German regions. It is the first attempt to combine all perspectives involved comprehensively. The combination of the chosen quantitative and qualitative methods seems to fit the research questions. They can complement each other and compensate deficiencies. Furthermore, we will get in-depth information of barriers and facilitators of implementation. A key challenge in this study is to develop useful recommendations. Therefore, after completing the first draft, the recommendations will be returned to the main actors in the healthcare conferences of the project regions. In summary, we expect to get a broad insight into the topic in order to develop comprehensive recommendations for creating and implementing low-threshold support services. A limitation of the study is that the questionnaires will be just pretested according to the feasibility and understanding but no comprehensive tests of reliability and validity can be conducted within this study. For further studies with an non-descriptive but an evaluative focus this is necessary.

The information can be used in the practical work for improving existing services but also for creating new ones. The work can also be useful for German policymakers when deciding on new regulations concerning low-threshold support services and on which initiatives are reasonable to finance. In general, the results contribute to developing health and social care structures in Germany.

There are structural differences in the care of people with dementia between Germany and other countries. However, it can be assumed that key issues, such as accessibility, are similar when these types of services are implemented. Therefore, this study can provide helpful information, also for other countries, on how these key issues are perceived by the services users and providers as well as which aspects future research should focus on.

## Abbreviations

FCG, Family caregiver; PWD, Person with dementia; V, Volunteer; C, Coordinator; IR, Institution representative.

## Competing interests

The authors declare that they have no competing interests.

## Authors’ contributions

IH had the initial idea for the study and will carry out the data collection and analysis. She drafted the manuscript. SB helped designing the study and will supervise it. BH helped designing the study and will accompany the data collection and analysis. SB and BH read and commented on the draft intensively. All authors approved the final manuscript.

## Authors’ information

IH is a scientific staff member of the working group care structures of the German Center for Neurodegenerative Diseases (DZNE) Witten. BH is the head of the working group care structures and SB is the speaker of the DZNE Witten and she holds the chair of Epidemiology-Nursing Science at the School of Nursing Science at the Witten/Herdecke University.
